# Detecting Blastocyst Components by Artificial Intelligence for Human Embryological Analysis to Improve Success Rate of In Vitro Fertilization

**DOI:** 10.3390/jpm12020124

**Published:** 2022-01-18

**Authors:** Muhammad Arsalan, Adnan Haider, Jiho Choi, Kang Ryoung Park

**Affiliations:** Division of Electronics and Electrical Engineering, Dongguk University, 30 Pildong-ro 1-gil, Jung-gu, Seoul 04620, Korea; arsal@dongguk.edu (M.A.); adnanhaider@dgu.ac.kr (A.H.); choijh1027@dongguk.edu (J.C.)

**Keywords:** blastocyst, embryological analysis, in vitro fertilization, deep learning, sprint convolutional block

## Abstract

Morphological attributes of human blastocyst components and their characteristics are highly correlated with the success rate of in vitro fertilization (IVF). Blastocyst component analysis aims to choose the most viable embryos to improve the success rate of IVF. The embryologist evaluates blastocyst viability by manual microscopic assessment of its components, such as zona pellucida (ZP), trophectoderm (TE), blastocoel (BL), and inner cell mass (ICM). With the success of deep learning in the medical diagnosis domain, semantic segmentation has the potential to detect crucial components of human blastocysts for computerized analysis. In this study, a sprint semantic segmentation network (SSS-Net) is proposed to accurately detect blastocyst components for embryological analysis. The proposed method is based on a fully convolutional semantic segmentation scheme that provides the pixel-wise classification of important blastocyst components that help to automatically check the morphologies of these elements. The proposed SSS-Net uses the sprint convolutional block (SCB), which uses asymmetric kernel convolutions in combination with depth-wise separable convolutions to reduce the overall cost of the network. SSS-Net is a shallow architecture with dense feature aggregation, which helps in better segmentation. The proposed SSS-Net consumes a smaller number of trainable parameters (4.04 million) compared to state-of-the-art methods. The SSS-Net was evaluated using a publicly available human blastocyst image dataset for component segmentation. The experimental results confirm that our proposal provides promising segmentation performance with a Jaccard Index of 82.88%, 77.40%, 88.39%, 84.94%, and 96.03% for ZP, TE, BL, ICM, and background, with residual connectivity, respectively. It is also provides a Jaccard Index of 84.51%, 78.15%, 88.68%, 84.50%, and 95.82% for ZP, TE, BL, ICM, and background, with dense connectivity, respectively. The proposed SSS-Net is providing a mean Jaccard Index (Mean JI) of 85.93% and 86.34% with residual and dense connectivity, respectively; this shows effective segmentation of blastocyst components for embryological analysis.

## 1. Introduction

**Figure 1:**
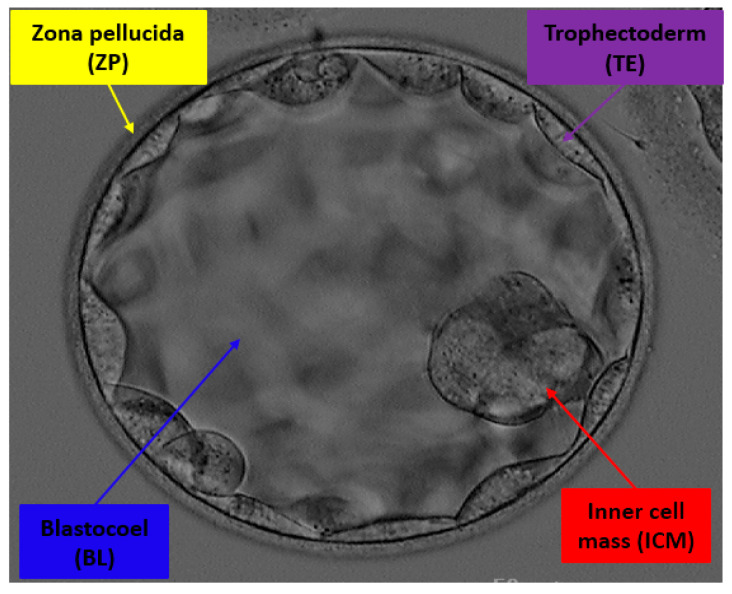
Example of blastocyst microscopic image with components ZP, TE, BL, and ICM, whose specific morphologies are considered to determine the viability of an embryo for IVF.

Infertility is a major clinical condition and a serious concern that affects 8–12% of couples, accounting for approximately 80 million couples worldwide [1]. The infertility rate has continuously grown across the globe, and even some West African communities are approaching an infertility rate of 50% [2]. Estimates indicate that 6.1 million people are affected by infertility in the United States, and only half of them are undergoing fertility-related treatments [3]. In vitro fertilization (IVF) is an effective and widespread form of assisted reproductive scheme used to treat infertility. IVF consists of a manual fertilization procedure in which embryos are cultured in an incubator for 3–5 days until they reach the blastocyst stage [4]. The best embryos (blastocysts) are selected based on valuable morphological attributes and transferred back to the patient’s uterus [5,6]. In the past, multiple blastocysts were transferred to increase the chance of pregnancy. However, it resulted in multiple pregnancies and other gestational issues in mothers and babies. Therefore, single viable blastocyst transfer is recommended to reduce the risk of multiple pregnancies [7,8]. Traditionally, the viability of the embryo is manually tested by careful observation of the morphological characteristics of blastocyst components by expert embryologists with a specific grading system using time-lapse imaging [9]. As shown in Figure 1, zona pellucida (ZP), trophectoderm (TE), inner cell mass (ICM), and blastocoel (BL) are essential parts of the blastocyst whose specific morphologies significantly contribute to pregnancy [10]. ZP is a protective glycoprotein layer that encapsulates the oocyte and has an important role in sperm-egg binding. The thickness of ZP is strongly related to IVF success [11], and it decreases as the embryo reaches the blastocyst stage. TE is a coating of cells that has a vital role in the creation of fluid to form the placenta. TE morphology and quality are highly correlated with embryo viability [12]. BL is a fluid cavity formed when an embryo is formed as a blastocyst on the fifth day, and this ICM is positioned on one side of the blastocyst. BL creation and morphology are correlated with IVF success [13]. Blastocyst quality also depends on ICM, which is the cluster mass of cells that results in the structure of the fetus [14]. The morphometric assessment of the blastocyst is an important objective of an embryologist, as the viability and the potential of the subject embryo depend on the characteristics of its components collectively [15]. According to Harada et al. [16], to increase the implantation rate (IVF success rate), blastocyst quality assessment is extremely important. ICM and TE are essential elements of the blastocyst that are observed during early cleavage. The formation and quality of the blastocyst can be monitored by the formation of the BL when the fluid fills the embryo completely. Manual assessment of blastocyst components is a crucial task that involves careful observation by embryologists, and this process can be automated using artificial intelligence (AI)-based algorithms.

In recent years, automated methods have been implemented to evaluate the viability and characteristics of blastocysts to improve the overall pregnancy rate by IVF. Although there are very few publicly available datasets on this topic, there are still several methods that use general image processing schemes or advanced deep learning-based methods. Conventional image processing-based methods use specific thresholds and parameters to detect features; therefore, it is not possible to detect multiple classes from an image using similar parameters. Wong et al. presented a particle filter-based tracking method for the day-by-day image analysis of zygotes for IVF [17]. Singh et al. proposed automatic segmentation of TE in microscopic images for embryological analysis. Specifically, they used Retinex filtering as preprocessing of the image, where the level-set method was used for segmentation of the TE with morphological post-processing [18]. Saeedi et al. presented an image-processing-based automatic scheme for the segmentation of TE and ICM. They used the biological characteristics with texture properties using watershed transform, and the ICM and TE regions were identified using physical maps from the histogram [19]. Filho et al. presented a semiautomatic method for the evaluation of blastocysts. They used the ellipse fitting method for the inner boundary of the ZP, and the outer boundary was identified by intensity-based thresholding. TE segmentation was performed using the level-set algorithm, where the ICM was segmented again using the variational level-set algorithm [20].

Learning-based methods can detect blastocyst components in a multiclass scenario. Starting from machine learning-based methods, Zaininovic et al. collected methods that are used for automatic grading of the embryo grading and assessment using image-level labeling approaches [4]. Bori et al. presented an artificial neural network (ANN)-based approach for the analysis of embryo morphology. In detail, they used image normalization and contrast adjustment as preprocessing. The segmentation of the components was conducted by Hough transformation and region analysis separately, and texture analysis was performed by ANN using 26 mathematical variables created by measuring the area of each component [10]. Kheradmand et al. proposed a neural network-based approach to detect ZP, TE, and ICM areas in blastocyst images. They used preprocessing and edge detection to detect the components [21]. A similar group presented a deep learning-based segmentation method to detect ICM from blastocyst images. They used a 16-layered fully convolutional network, wherein the preprocessing step partially contained the blastocyst, and the background pixels were removed using the ZP boundary [22]. Rad et al. presented a stacked dilated U-Net architecture to segment the ICM from the background for embryological analysis. They utilized an optimized design by choosing the kernel size, depth of the network, and dilation rate for better segmentation performance [23]. Considering the efficacy of semantic segmentation, Rad et al. presented another deep learning-based architecture to detect multiclass ZP, TE, BL, and ICM from blastocyst images. They used the backbone ResNet-50 network in the encoder part, and cascaded atrous pyramid pooling was used to incorporate multiscale features. Dense progressive sub-pixel upsampling was used inside the decoder [24]. The same group presented an ensemble-based boosting network to detect a single-class ZP. In detail, they utilized a patch-based approach with sizes of 7 × 7, 11 × 11, 15 × 15, 19 × 19, and 23 × 23 pixels to input the hierarchical network. Self-supervised image-specific refinement was utilized to improve segmentation performance [25]. Huang et al. used a deep neural network on time-lapse images for the analysis of human blastocysts. The segmentation task was conducted using an optimized U-Net, which is considered good for medical image segmentation [26]. TE segmentation was performed using the Inception U-Net architecture for embryological analysis. A generative approach was used for synthetic image creation, and the original inception module was modified by incorporating dilated convolutions [27]. Wang et al. utilized the VGG-16 architecture for the classification of blastocyst images using a private dataset. They used the VGG-16 ensemble and MobileNetV2 ensemble with different combinations to improve classification performance [28]. Most of the previous studies on blastocyst analysis considered only a single class, and they proposed deep architectures that consume a large number of trainable parameters. In this study, we propose a novel shallow architecture SSS-Net that provides low cost-robust segmentation for embryological analysis.

In the last decade, few researchers have focused on automatic embryo selection procedures using machine learning [29]. From Figure 1, the microscopic image of the blastocyst has non-uniform illumination, and the gray levels are very close to each other. More specifically, ICM and TE appear very similar. The detection of similar structures with conventional image processing schemes is very difficult and requires parameter tuning repeatedly with the change in image acquisition. AI has the potential to assist embryology in the selection of the best-fit embryo transfer for IVF. Automated AI assessment of the embryo increases the efficacy of viable blastocyst assortment for implantation [30,31]. Very few researchers have focused on automated methods for the detection of blastocyst components. Most of these methods provide lower accuracy and require significant computational power. Considering the advancement of deep learning and its benefits in computer-aided diagnosis [32,33,34], this study proposes a novel sprint semantic segmentation network (SSS-Net) that accurately detects the blastocyst components (ZP, TE, BL, and ICM) for embryological analysis and improves the success rate of IVF.

This study aims to provide a platform to embryologists where the blastocyst component morphology can be provided with accuracy. The accurate detection of these components (ZP, TE, BL, and ICM) in a multiclass scenario is important for collective embryological analysis. As stated above, the blastocyst morphological analysis can lead to a single viable blastocyst transfer for safe in vitro fertilization. Moreover, this study is a step towards the development of low-cost automatic embryo morphology assessment using handheld devices. SSS-Net is an accurate shallow semantic segmentation network that uses sprint convolutional blocks (SCBs), which are specifically designed to provide accurate segmentation using a low number of trainable parameters and floating-point operations. The SCB considers asymmetric kernel and depth-wise separable convolutions in a unique design that allows the network to perform better with reduced computational cost. The main contributions of this study are as follows:

- Multiclass semantic segmentation architecture that segments ZP, TE, BL, and ICM from the background without preprocessing.
- SCB uses asymmetric kernel-based convolutions in combination with depth-wise separable convolutions to reduce floating-point operations. Low-cost shallow architecture with an overall 4.04 million trainable parameters and 28 Giga floating-point operations per second (GFLOPS).
- The SSS-Net provides high segmentation performance, and the output of the network can be used to observe morphometric properties of the blastocyst components for embryological analysis and blastocyst viability assessment.
- Our trained networks and codes are publicly available for comparison [35].

The remainder of this paper is organized as follows. In Section 2, we present the proposed method. In Section 3 and Section 4, we present results, and discussion, respectively. Finally, we provide the conclusions in Section 5.

## 2. Material and Methods

### 2.1. Datasets

**Figure 2:**
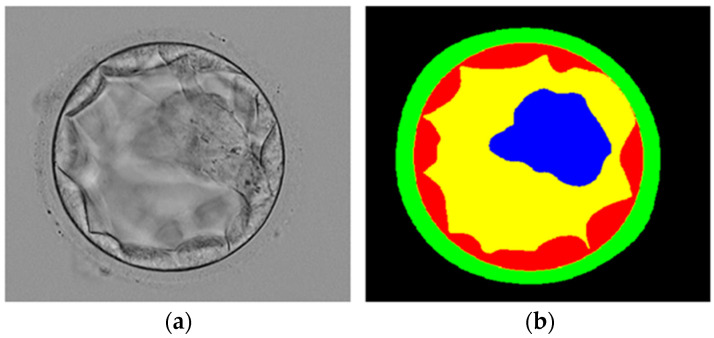
Example blastocyst microscopic image: (**a**) original image, (**b**) manual expert embryologist label image.

In this study, we utilized the blastocyst image dataset introduced in [19], which is the only blastocyst image dataset publicly available. The dataset included 235 Hoffman Modulation Contrast (HMC) microscopic blastocyst images captured by an Olympus IX71 inverted microscope using the Research Instrument Cronus 4 software (Falmouth, England). All images were captured at magnifications of 1.6× and 20× and objective lens. These images are from the different patients who were treated at Pacific Center for Reproduction Canada between 2012 to 2016, images are randomly chosen with a good focus on both TE and ICM. These blastocyst images were manually labeled by expert embryologists for blastocyst components. The labeled images, called ground truth (GT), were collectively made available by [19] for research purposes with the approval of the Canadian Research Ethics Board on 24 May 2017. We followed the same train-test split criteria of the learning-based method [24], and out of 235 images, 85% (200) images were used for training and 15% (35) for testing. To fairly compare our method with existing learning-based methods [24,36,37,38,39] which used the same experimental dataset as ours, we followed the same train-test split criteria mentioned by [24]. Figure 2 presents an example blastocyst image with an expert embryologist label image.

### 2.2. Method

#### 2.2.1. Summary of Proposed Method

**Figure 3:**
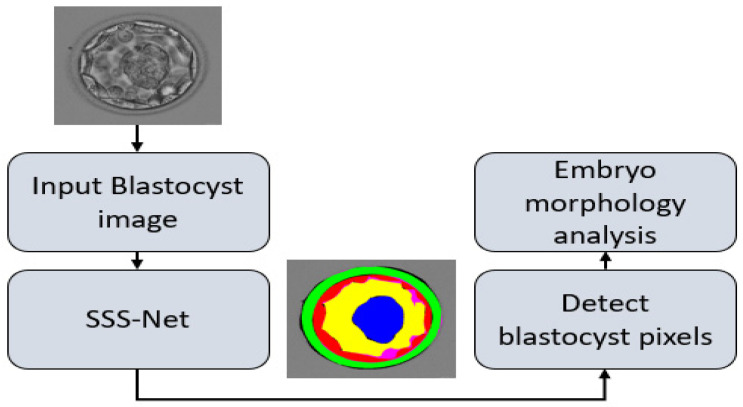
The overall workflow of the proposed method to detect blastocyst components for embryological analysis. Abbreviations: SSS-Net, sprint semantic segmentation network.

This study presents a shallow semantic segmentation architecture to detect blastocyst components for embryological analysis. Figure 3 presents the overall workflow of the proposed method. The proposed SSS-Net avoids expensive preprocessing schemes to enhance image contrast. SSS-Net takes the original blastocyst image in raw form without preprocessing, applies SCBs inside the encoder to extract valuable discriminative features, and utilizes a shallow upsampling block as a decoder. At the output, SSS-Net provides a five-channel mask, where each channel represents a specific class of ZP, TE, BL, ICM, and background. These output masks contain each blastocyst component pixel marked with ‘1’ and other pixels with ‘0,’ and can be used to analyze the morphology of each component to assess blastocyst viability.

#### 2.2.2. Structure of Proposed Encoder Block

**Figure 4:**
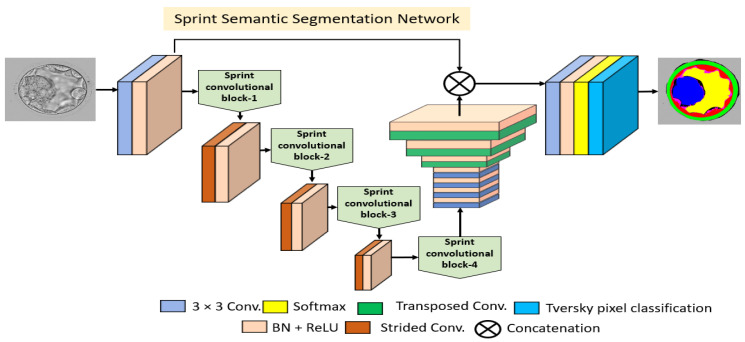
The architecture of proposed SSS-Net. 3 × 3 Conv. and Transposed Conv. represent convolution layers with 3 × 3 kernel and transposed convolution, respectively. In addition, BN, ReLU, and Strided Conv. represent batch normalization, rectified linear unit, and strided convolution, respectively.

**Figure 5:**
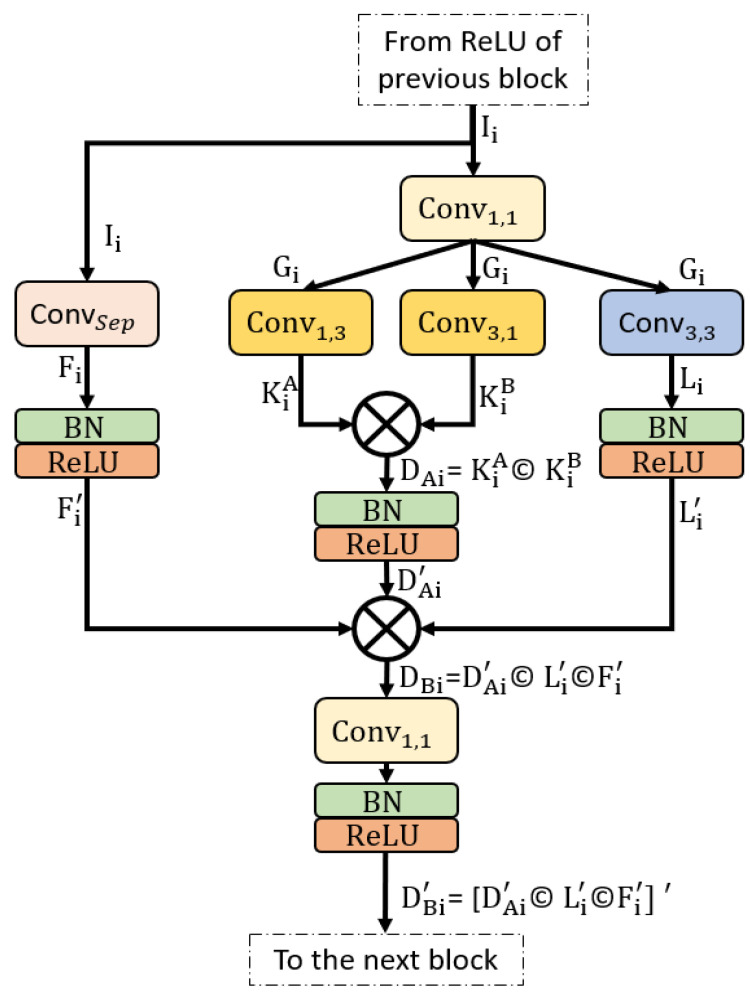
Sprint convolutional block connectivity pattern with two times feature concatenation. Abbreviations: ${\mathrm{Conv}}_{Sep}$, separable convolution; ${\mathrm{Conv}}_{1,\;1}$, 1 × 1 convolution; ${\mathrm{Conv}}_{1,\;3}$, asymmetric kernel 1 × 3 convolution; ${\mathrm{Conv}}_{3,\;1}$, asymmetric kernel 3 × 1 convolution; ${\mathrm{Conv}}_{3,\;3}$, 3 × 3 convolution; BN, batch normalization; ReLU, rectified linear unit.

Conventional semantic segmentation architectures have an encoder that is the same as the decoder. If the encoder consumes many trainable parameters, these parameters are doubled when using a similar decoder [36,40]. SSS-Net is a shallow semantic segmentation architecture that consumes a low number of trainable parameters, and the upsampling part uses few transposed convolutions. Figure 4 shows the layer-by-layer schematic diagram for SSS-Net, and Figure 5 shows a schematic of the proposed SCB. Because extensive usage of pooling layers causes loss of important spatial information, which may result in performance deterioration [41], we utilized three strided convolutions to reduce the size feature map inside the network with learned weights. Dense connectivity covers the feature transfer impedance problems that exist in conventional networks [42]. As shown in Figure 4, the overall encoder uses four SCBs (SCB-1–SCB-4), where each SCB concatenates different features. According to Figure 5, each SCB point-wise convolution ${\mathrm{Conv}}_{1,1}$ receives the $I_{i}$ feature from the rectified linear unit (ReLU) of the previous SCB, and the separable convolution ${\mathrm{Conv}}_{Sep}$ receives the same $I_{i}$ in parallel as input. The point-wise features $G_{i}$ from ${\mathrm{Conv}}_{1,1}$ are parallelly provided to two asymmetric kernel-based convolutions (${\mathrm{Conv}}_{1,3}$, ${\mathrm{Conv}}_{3,1}$) and a normal convolution (${\mathrm{Conv}}_{3,3}$), which output $K_{i}^{A}$, $K_{i}^{B}$, and $L_{i}$, respectively. The features after asymmetric kernel convolutions $K_{i}^{A}$, $K_{i}^{B}$ are concatenated to provide $D_{\mathrm{Ai}}$ given by (1). Subsequently, this feature $D_{\mathrm{Ai}}$ passes through batch normalization (BN) and ReLU to produce $D_{\mathrm{Ai}}^{\prime }$ ready to combine again with other features.

(1) $$D_{\mathrm{Ai}}=K_{i}^{A}©\;K_{i}^{B}$$

Here, © shows the depth-wise concatenation between features $K_{i}^{A}$ and $K_{i}^{B}$. The outputs of ${\mathrm{Conv}}_{Sep}$ and ${\mathrm{Conv}}_{3,3}$ are $F_{i}$ and $L_{i}$, which alter to $F_{i}^{\prime }$ and $L_{i}^{\prime }$, respectively, after BN and ReLU operations. The features from ${\mathrm{Conv}}_{Sep}$ and ${\mathrm{Conv}}_{3,3}$ ($F_{i}^{\prime }$ and $L_{i}^{\prime }$) are combined with the asymmetric convolution feature $D_{\mathrm{Ai}}^{\prime }$ to create an enhanced feature $D_{\mathrm{Bi}}$ given by (2). This $D_{\mathrm{Bi}}$ feature involves $F_{i}^{\prime }$ spatial information imported from the previous block. This $D_{\mathrm{Bi}}$ feature alters to $D_{\mathrm{Bi}}^{\prime }$ after one bottleneck ${\mathrm{Conv}}_{1,1}$ at the end of candidate SCB and BN. The ReLU operation is given by (3).

(2) $$D_{\mathrm{Bi}\;}={\;D}_{\mathrm{Ai}}^{\prime }©{\;L}_{i}^{\prime }{©F}_{i}^{\prime }$$

(3) $$D_{\mathrm{Bi}}^{\prime }\;={[D}_{\mathrm{Ai}}^{\prime }{©\;L}_{i}^{\prime }{©F}_{i}^{\prime }]{\;}^{\prime }$$

Here, © shows the depth-wise concatenation among features $D_{\mathrm{Ai}}^{\prime }$, $L_{i}^{\prime }$, and $F_{i}^{\prime }$. Feature $D_{\mathrm{Bi}}^{\prime }$ is the final feature from the candidate SCB that aggregates three different features, and it is available for the next SCB. The encoder layers and layer-wise feature map size details are listed in Table S1.

#### 2.2.3. Structure of Proposed Decoder Block

Unlike conventional semantic segmentation architectures, SSS-Net has a different shallow decoder block that uses only three transposed convolutions. The feature $D_{\mathrm{Bi}}^{\prime }$ in the last SCB size is halved three times, and the three transposed convolutions are used to upsample three times to match the feature with the original input size. SSS-Net is a shallow architecture that uses a few layers inside the decoder to upsample. As shown in Figure 4, the decoder uses four convolutions as a bridge between the encoder and transposed convolution for deep feature learning. Three transposed convolutions upsample the image back to the original and provide these features to the final pixel classification block. The decoder layers and layer-wise feature map size details are listed in Table S1. The pixel classification block consists of a convolution whose filters are set to the number of classes, and the image pixels are classified using a pixel classification layer that utilizes Tversky loss [43] to address the class imbalance and provide better segmentation. The Tversky loss ($T_{\mathrm{Loss}}$) is given by (4).

(4) $$T_{\mathrm{Loss}\;}=\frac{\sum_{i=1}^{N}P_{b}^{i}{\;G}_{b}^{i}+\;\omega }{\sum_{i=1}^{N}P_{b}^{i}{\;G}_{b}^{i}+\;\omega \;+\;\alpha \;\sum_{i=1}^{N}P_{\mathrm{nb}}^{i}{\;G}_{b}^{i}+\;\beta \;\sum_{i=1}^{N}P_{b}^{i}{\;G}_{\mathrm{nb}}^{i}+\;\omega }$$

where $P_{b}^{i}$ and $G_{b}^{i}$ are the probabilities of the pixel belonging to the blastocyst and non-blastocyst components, respectively. $G_{b}^{i}$ an $G_{\mathrm{nb}}^{i}$ are the pixels that belong to a blastocyst and to a non-blastocyst component in the ground truth, respectively. α and β are the components that can set the trade-off between false positives and false negatives by changing the values between (0,1), given that α + β = 1. In our experiments, α=0.7 and β=0.3 were used. ω is a component used to avoid division by zero.

#### 2.2.4. Experimental Environment and Data Augmentation

The proposed SSS-Net was implemented using an NVIDIA RTX 3080 (Santa Clara, CA, USA) [44] GPU on a desktop computer using an Intel^®^ Core-i7-3770K (Santa Clara, CA. USA) processor with 28 GB of RAM. The network was implemented on MATLAB R2021a [45] using Microsoft Windows 10 (Washington, DC, USA). To train the proposed SSS-Net initial learning rate 0.0001, Adam optimizer [46], Epsilon 0.000001, global-l2 normalization hyperparameters are used, where the network is trained for 11200 iterations with a mini-batch size of 20 images.

To appropriately train a deep learning network, sufficient training data are required. In the case of medical imaging for disease analysis, it is very difficult to obtain massive data. Therefore, data augmentation (synthetic image generation) is required to increase the number of training images. In this study, we utilized data augmentation schemes using image operations, including image flipping, image translation, and rotations. Further details of this augmentation are described in [33]. The SSS-Net is a segmentation network; therefore, during the augmentation process, the same image operation is applied to the image and the GT to create the training data. In detail, from 200 training images, we created 3200 image-GT pairs using data augmentation.

#### 2.2.5. Ablation Study

Feature empowerment is a scheme in which better segmentation performance is achieved by introducing skip connections. These skip connections import the edge information from the initial layers to reduce feature deterioration effects. Thus, the SSS-Net uses the concatenation of these imported features for better performance. As both residual and dense connectivity is commonly used to address the vanishing gradient problem, they were used in an ablation study conducted for SSS-Net, as shown in Figure 4. In detail, the features from the skip connection were element-wise added in one case and depth-wise concatenated in the other case. As shown in Table 1, SSS-Net with dense feature concatenation provides high segmentation performance compared with that by SSS-Net with residual connectivity.

## 3. Results

### 3.1. Evaluation of Proposed Method

At the testing phase, the proposed SSS-Net provides five binary masks for ZP, TE, BL, ICM, and background, respectively, with a representation of desired class with “1” and the non-desired class with “0”. We utilized versatile Jaccard index (JI) measure to evaluate our method which is similarly used by [24]. JI is given by (5).

(5) $$\mathrm{JI}=\frac{\mathrm{TP}}{\mathrm{TP}\;+\;\mathrm{FP}\;+\;\mathrm{FP}}$$

where TP represents true positive (the pixel predicted as a blastocyst component is a blastocyst component in GT). FN represents a false negative (the pixel predicted as the background is a blastocyst pixel in GT). FP represents a false positive pixel (the pixel predicted as a blastocyst pixel is listed as a background pixel in GT).

### 3.2. Comparison of Proposed Method with Existing Methods

This section offers a numerical comparison of the proposed method with that of state-of-the-art methods based on JI given by (4) and the number of trainable parameters. It can be observed from Table 2 that the proposed SSS-Net is based on asymmetric filtered convolutions that help to reduce the number of trainable parameters. The SSS-Net consumes 4.04 million trainable parameters, which is considerably small compared to the number of parameters consumed by existing methods. Table 2 also reveals that SSS-Net (residual) performs better than all existing approaches, with a mean JI of 85.93%. SSS-Net with dense feature concatenation performs better than the methods listed in Table 2. The dense connectivity provided better segmentation performance with a mean JI of 86.34%. The boundary of the ZP is crucial because of the low contrast compared to the background, where the existing methods do not perform better for this class, considering that the SCB captures the complex distinctive features of the ZP class. These features are combined with low-level spatial information using dense connectivity, which increases the segmentation performance of the ZP class.

The results of all the previous methods [24,36,37,38,39] are taken from [24]. The methods of UNet-Baseline [36], TernausNet U-Net [37], PSP-Net [38], and DeepLab V3 [39] are those that were designed for different tasks (other than blastocyst segmentation), but these methods are implemented by [24] using the same train-test criteria and protocols. Our proposed SSS-Net Residual and SSS-Net Dense are following the same experimentation criteria defined by [24]. Abbreviations: SSS-Net, sprint semantic segmentation network; UNet, U-shaped network; PSP-Net, pyramid scene parsing network; Blast-Net, blastocyst network; ZP, zona pellucida; TE, trophectoderm; BL, blastocoel; ICM, inner cell mass; JI, Jaccard index.

### 3.3. Visual Results of Proposed Method for Blastocyst Component Detection

**Figure 6:**
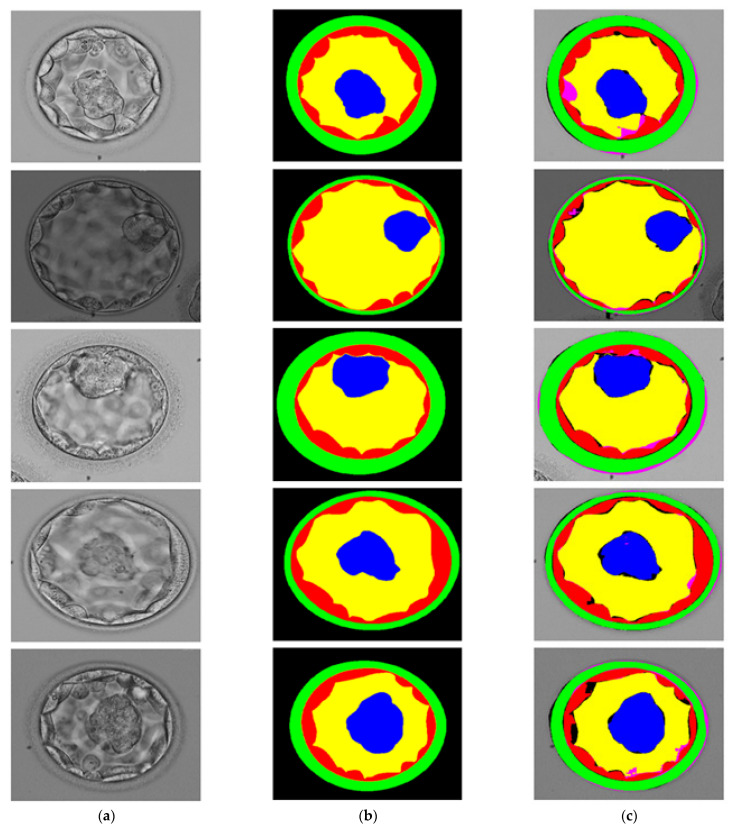
Visual Results of Proposed SSS-Net for blastocyst component detection: (**a**) Original Input Image, (**b**) Expert Annotation, and (**c**) Predicted Image Mask by SSS-Net.

The proposed SSS-Net is a multiclass segmentation network. It can be noticed from Table S1, the final feature map from the SSS-Net has five channels, and each channel represents a single-class mask from ZP, TE, BL, ICM, and background. Each mask represents the desired class pixels with ‘1’ and other pixels with ‘0′. Figure 6 shows the visual results of blastocyst image segmentation using the proposed SSS-Net with the corresponding GT images. The green, red, yellow, blue, black/no-colors (GT/predicted) represent the ZP, TE, BL, ICM, and background classes, respectively. The pink color in the predicted image presents a false negative for each class, which indicates a disagreement between GT and the predicted mask with GT = 1 and predicted mask = 0. The black color in the predicted image represents the false positive pixels, which shows a disagreement between the GT and the predicted mask with GT = 0 and predicted mask = 1.

## 4. Discussion

Table 2 presents the numerical comparison of the proposed method with currently available state-of-the-art methods. It can be noticed from Table 2 that the crucial zona pellucida (ZP) region is effectively detected by SSS-Net with a mean JI of 82.88% and 84.51 with residual and dense connectivity, respectively. The existing state-of-the-art segmentation methods [24,36,37,38,39] are not performing well for ZP. Considering inner cell mass (ICM), the SSS-Net with residual connectivity is providing a mean JI of 84.94% which is much more than 79.03% by [36], and 77.58% by [37]. Considering all ZP, TE, BL, ICM, and background classes SSS-Net with dense connectivity and residual connectivity provided an overall mean JI of 86.34% (first place), and 85.93% (second place), respectively. The proposed SSS-Net is just consuming 4.04 million trainable parameters which are the least among all the methods available in Table 2. There is no description of the decisions made by the deep networks, which are considered as a black box with no explanation. Gradient weighted class activation mapping (Grad-CAM) [47] provides the key features that are involved in the decision-making of SSS-Net. The details about Grad-CAM can be found in Section S1 and Figure S1.

### 4.1. Principal Findings

As explained in Section 2.2.1, the SSS-Net outputs five masks, and each mask represents one candidate class. These masks can be used to accurately detect the boundaries of blastocyst components. The morphology of the blastocyst components (ZP, TE, BL, and ICM) is very important to verify their viability. The success of IVF depends on the specific proportion and morphological properties of these components [11,12,13,14,16]. Figure S2 shows an example image with the detection masks of ZP, TE, BL, and ICM, which have accurately detected boundaries that can be individually and collectively analyzed by the expert embryologist for viability. To provide better analysis, the numerical proportions and areas of these detected morphologies can be provided for detailed observation. Moreover, the position of these components can be a beneficial tool for analyzing the location properties of blastocysts. The thickness of the ZP is extremely important for pregnancy by IVF and can be estimated using the predicted ZP mask. BL is the component formed on the 5th day when the embryo is converted into a blastocyst. The BL predicted mask can represent the formation of the blastocyst, which can be transferred to the uterus for successful fertilization

### 4.2. Limitations and Future Work

The availability of medical images is a serious concern, and it limits the performance of learning-based methods. Although SSS-Net is providing superior segmentation performance for all blastocyst components, still there are a few limitations of the current study. The datasets used in this study are the only publicly available multiclass datasets, and the deep-learning networks require more data to get sufficiently trained. The data augmentation procedure is essentially required to create synthetic images for successful training with a low number of images; therefore, the data augmentation is used to synthetically generate the images for better training of the proposed method. Moreover, as a deep learning method, the training requires the labeled images which are from the expert embryologist. The proposed method’s accuracy is highly related to the precision of the training image by the embryologist. In the future, we intend to collect more embryological data and to reduce the model cost more to enhance overall system capability.

## 5. Conclusions

In this study, a novel semantic segmentation architecture for multiclass blastocyst components for embryological analysis is presented. The proposed SSS-Net is a shallow architecture that uses asymmetric kernel-based convolutions and depth-wise separable convolutions in an SCB. Each SCB has feature empowerment, which allows it to learn valuable features to accurately segment blastocyst components. The proposed SSS-Net detects ZP, TE, BL, and ICM in a multiclass manner, and these detected masks are accurate for embryological analysis. The experimental results confirmed that our proposal provides promising segmentation performance with a Jaccard Index of 82.88%, 77.40%, 88.39%, 84.94%, and 96.03% for ZP, TE, BL, ICM, and background, with residual connectivity, respectively. It also provides a Jaccard Index of 84.51%, 78.15%, 88.68%, 84.50%, and 95.82% for ZP, TE, BL, ICM, and background, with dense connectivity, respectively. The proposed SSS-Net is providing a mean Jaccard Index (Mean JI) of 85.93% and 86.34% with residual and dense connectivity, respectively The proposed method can be used to verify the morphological properties of blastocysts for successful IVF procedures. In the future, we will extend this work to other medical image analyses and attempt to optimize this network for mobile platforms.

## Figures and Tables

**Table 1:** Ablation study of proposed SSS-Net.

Method	No. of Parameters	Mean JI	Model Size	GFLOPS
SSS-Net (Residual)	4.04 M	85.93	15.0 MB	28
SSS-Net (Dense)	4.04 M	86.34	14.5 MB	28

Abbreviations: SSS-Net, sprint semantic segmentation network; JI, Jaccard index; GFLOPS, Giga floating-point operations per second.

**Table 2:** Numerical Comparison of Proposed SSS-Net with the State-of-the-Art Schemes.

Method	No. of Parameters	ZP	TE	BL	ICM	Background	Mean JI
UNet-Baseline [36]	31.03 M	79.32	75.06	79.41	79.03	94.04	81.37
TernausNet U-Net [37]	10 M	80.24	76.16	78.61	77.58	94.50	81.42
PSP-Net [38]	35 M	80.57	74.83	79.26	78.28	94.60	81.51
DeepLab V3 [39]	40 M	80.84	73.98	78.35	80.60	94.49	81.65
Blast-Net [24]	25 M	81.15	76.52	80.79	81.07	94.74	82.85
SSS-Net Residual (Proposed)	4.04 M	82.88	77.40	88.39	84.94	96.03	85.93
SSS-Net Dense (Proposed)	4.04 M	84.51	78.15	88.68	84.50	95.82	86.34

## Data Availability

Not applicable.

## Supplementary Materials

The following supporting information can be downloaded at https://www.mdpi.com/article/10.3390/jpm12020124/s1, containing: Table S1, Feature map size for the proposed SSS-Net; Section S1, Grad-CAM explanation of the proposed method; and Figure S1, Grad-CAM visualization of SSS-Net, Three examples are shown in three rows: (a) input blastocyst; (b) GT images; and (c–f) images taken from Conv-F4-3_3, Tconv-F1, Tconv-F2, and Tconv-F3 of Table S1, respectively; Figure S2, Predicted masks of blastocyst components for embryological analysis by SSS-Net: (a) input blastocyst image and (b) ZP, (c) TE, (d) BL, (e) ICM, and (f) combined predicted masks.
