# Supplementary material for: Detecting Blastocyst Components by Artificial Intelligence for Human Embryological Analysis to Improve Success Rate of In Vitro Fertilization
Source: J Pers Med. 2022 Jan 18;12(2):124. doi: 10.3390/jpm12020124 (PMC8877842; doi:10.3390/jpm12020124)
Supplement: Supplementary file 1 [file jpm-12-00124-s001.zip › jpm-1547185-supplementary.pdf]

Journal of Personalized Medicine

MDPI

Supplementary Materials

Table S1. Feature Map Size for the proposed SSS-Net.

Block	Layer	(K,K)/S,F	Output (width(height)2, number of channels)
Input	Conv-in	(3,3)/1,32	400^2,32
SCB-1	Sep-Conv1	(3,3)/1,32	400^2,32
	Conv-A-1_1*	(1,1)/1,16	400^2,16
	Conv-AS1*	(3,1)/1,8	400^2,8
	Conv-AS2*	(1,3)/1,8	400^2,8
	Cat-SCB1-A	-	400^2,16
	Conv-A-3_3	(3,3)/1,16	400^2,16
	Cat-SCB1-B	-	400^2,64
	Conv-A-1_1*	(1,1)/1,32	400^2,32
Reduction	Conv-Stride-1	(3,3)/2,64	200^2,64
SCB-2	Same as SCB-1	F×2	200^2,64
Reduction	Conv-Stride-2	(3,3)/2,128	200^2,128
SCB-3	Same as SCB-2	F×2	100^2,128
Reduction	Conv-Stride-3	(3,3)/2,256	50^2,256
SCB-4	Same as SCB-3	F×2	50^2,384
Final Block	Conv-F1-3_3	(3,3)/1,384	50^2,384
	Conv-F2-3_3	(3,3)/1,256	50^2,256
	Conv-F3-3_3	(3,3)/1,256	50^2,256
	Conv-F4-3_3	(3,3)/1,128	50^2,128
	Tconv-F1	(2,2)/1,64	100^2,64
	Tconv-F2	(2,2)/1,32	200^2,32
	Tconv-F3	(2,2)/1,32	400^2,32
	Cat-F	-	400^2,64
	Conv-Mask	(1,1)/1,5	400^2,5
Total number of trainable parameters = 4.04 Million			

Abbreviations: SCB, sprint convolutional block, Conv-in, input convolution, Sep-Conv1, separable convolution-1, Conv-A-1_1, 1 × 1 convolution-1, Conv-AS1, asymmetric kernel convolution-1, Conv-AS2, asymmetric kernel convolution-2, Cat-SCB1-A, sprint convolutional block-1 concatenation-1, Conv-A-3_3, 3 × 3 convoltuinal-1, Cat-SCB1-B, sprint convolutional block-1 concatenation-2, Conv-Stride- convolution with stride, Conv-F1-3_3, 3 × 3 convoltuinal-1 of final block, Conv-F2-3_3, 3 × 3 convoltuinal-2 of final block, Conv-F3-3_3, 3 × 3 convoltuinal-2 of final block, Conv-F4-3_3, 3 × 3 convoltuinal-4 of final block, Tconv-F1, transposed convolution-1 of final block, Tconv-F2, transposed convolution-2 of final block, Tconv-F3, transposed convolution-3 of final block, Cat-F, final concatenation, Conv-Mask, mask convolution, K, kernel size, S, stride, F, number of filters. Symbol "*" show that the layers are without batch normalization (BN) rectified linear unit (ReLU). The symbol "-" show that this information is not available for that specific layer.

Section. S1. Grad-CAM Explanation of the Proposed Method

The effective development of these networks requires knowledge of the key features in the image that are required to conclude the prediction of a specific class. The development principles may change according to the application and key features for better accuracy. The gradient-weighted activation (Grad-CAM) [47] is a scheme that enlightens the

beneficial features that are chosen by the neural network to predict the candidate class. Figure S1 shows the Grad-CAM of SSS-Net taken from the intermediate layers of the network as an example. Figure S1 (c)–(f) show the heat maps obtained from Conv-F4-3_3, Tconv-F1, Tconv-F2, and Tconv-F3 of Table S1 (Supplementary Materials), respectively. The following are important points that are concluded herein.

- Robust semantic segmentation architecture can provide accurate blastocyst component segmentation (ZP, TE, BL, ICM, etc.);
- The deep learning-based model can perform multiclass segmentation in one step, and does not require training of the network several times for different classes;
- To achieve semantic segmentation, a decoder that is the same as the encoder is not required. A shallow decoder can perform well (SSS-Net uses only three transposed convolutions for upsampling);
- The SCB helps to reduce the number of parameters by using asymmetric kernel convolutions and depth-wise separable convolutions. The SSS-Net consumes only 4.04 million trainable parameters;
- If the size of the feature map is significantly reduced (using multiple pooling layers), it can miss minor information. The SSS-Net downsamples the image three times with a final feature map size of 50 × 50, which is sufficient to represent useful spatial information;
- The initial layers contain spatial edge information. If this information is provided to the final layers of the network, the segmentation performance can be improved;
- Accurate segmentation of blastocyst components can ease the morphological analysis by the embryologist.

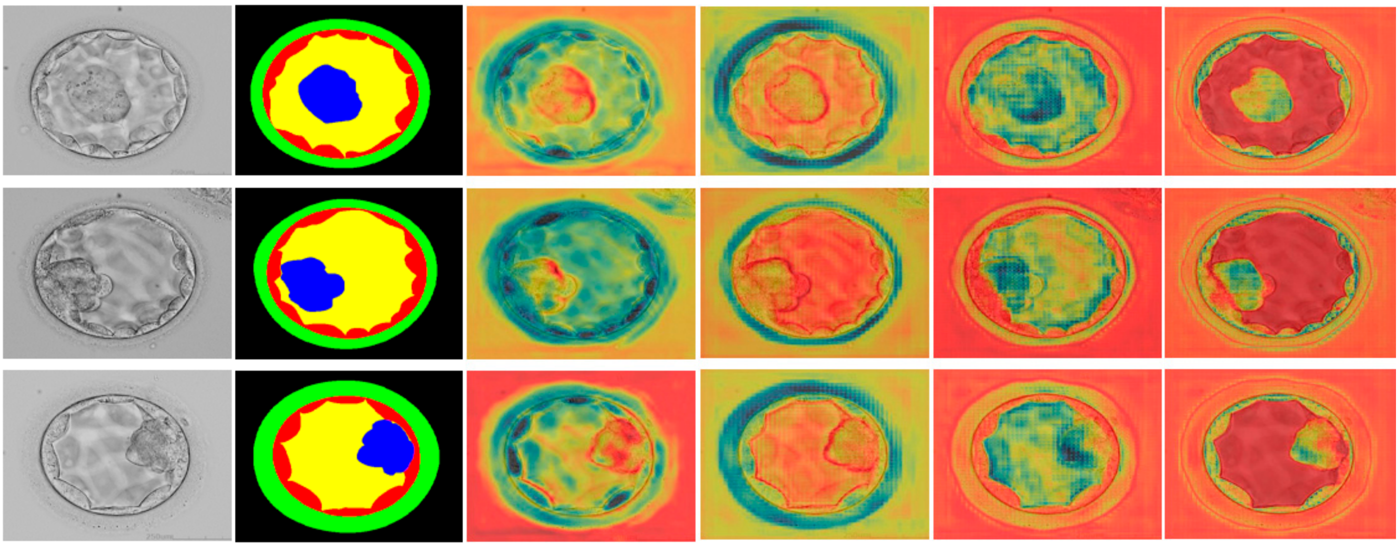

(a) **(b)** **(c)** **(d)** **(e)** **(f)**

Figure S1. Grad-CAM visualization of SSS-Net. Three examples are shown in three rows: (a) input blastocyst and (b) GT images. (c)-(f) Images taken from Conv-F4-3_3, Tconv-F1, Tconv-F2, and Tconv-F3 of Table S1 (Supplementary Materials), respectively. Abbreviations: Grad-CAM, gradient weighted class activation mapping, SSS-Net, sprint semantic segmentation network, GT, ground truth.

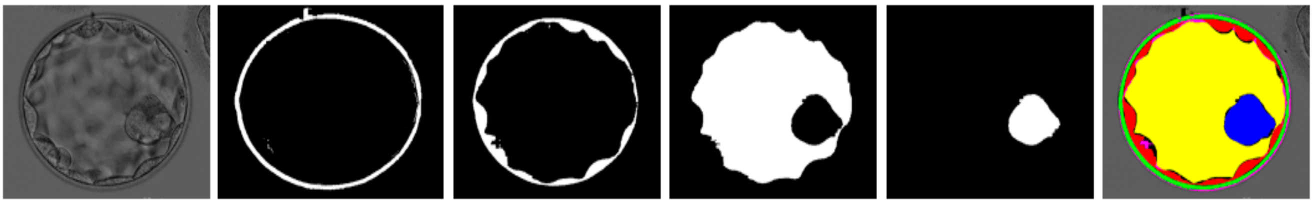

(a) (b) (c) (d) (e) (f)

Figure S2. Predicted masks of blastocyst components for embryological analysis by SSS-Net: (a) input blastocyst image and (b) ZP, (c) TE, (d) BL, (e) ICM, and (f) combined predicted masks. Abbreviations: SSS-Net, sprint semantic segmentation network, ZP, zona pellucida, TE, trophectoderm, BL, blastocoel, ICM, inner cell mass.
